# Primary care physicians’ knowledge and confidence in providing cancer survivorship care: a systematic review

**DOI:** 10.1007/s11764-023-01397-y

**Published:** 2023-05-12

**Authors:** Julien A. M. Vos, Barbara M. Wollersheim, Adelaide Cooke, Carolyn Ee, Raymond J. Chan, Larissa Nekhlyudov

**Affiliations:** 1 Department of General Practice, Amsterdam UMC, location University of Amsterdam, Meibergdreef 9, Amsterdam, the Netherlands; 2 Amsterdam Public Health, research programme Quality of Care, and Personalized Medicine, Amsterdam, the Netherlands; 3 Division of Psychosocial Research and Epidemiology, The Netherlands Cancer Institute, Antoni van Leeuwenhoek Hospital, Plesmanlaan 121, 1066 CX Amsterdam, The Netherlands; 4 MS1 at University of North Carolina School of Medicine, Chapel Hill, NC USA; 5 NICM Health Research Institute, Western Sydney University, Penrith, New South Wales Australia; 6 Caring Futures Institute, College of Nursing and Health Sciences, Flinders University, South, Adelaide, Australia; 7 Department of Medicine, Brigham and Women’s Hospital, Harvard Medical School, Boston, MA USA

**Keywords:** Primary health care, Cancer, Survivorship, Education, Evaluation

## Abstract

**Purpose:**

To systematically review existing literature on knowledge and confidence of primary care physicians (PCPs) in cancer survivorship care.

**Methods:**

PubMed, Ovid MEDLINE, CINAHL, Embase, and PsycINFO were searched from inception to July 2022 for quantitative and qualitative studies. Two reviewers independently assessed studies for eligibility and quality. Outcomes were characterized by domains of quality cancer survivorship care.

**Results:**

Thirty-three papers were included, representing 28 unique studies; 22 cross-sectional surveys, 8 qualitative, and 3 mixed-methods studies. Most studies were conducted in North America (*n* = 23) and Europe (*n* = 8). For surveys, sample sizes ranged between 29 and 1124 PCPs. Knowledge and confidence in management of physical (*n* = 19) and psychosocial effects (*n* = 12), and surveillance for recurrences (*n* = 14) were described most often. Generally, a greater proportion of PCPs reported confidence in managing psychosocial effects (24–47% of PCPs, *n*= 5 studies) than physical effects (10–37%, *n* = 8). PCPs generally thought they had the necessary knowledge to detect recurrences (62–78%, *n* = 5), but reported limited confidence to do so (6–40%, *n* = 5). There was a commonly perceived need for education on long-term and late physical effects (*n* = 6), and cancer surveillance guidelines (*n* = 9).

**Conclusions:**

PCPs’ knowledge and confidence in cancer survivorship care varies across care domains. Suboptimal outcomes were identified in managing physical effects and recurrences after cancer.

**Implications for Cancer Survivors:**

These results provide insights into the potential role of PCPs in cancer survivorship care, medical education, and development of targeted interventions.

**Supplementary Information:**

The online version contains supplementary material available at 10.1007/s11764-023-01397-y.

## Introduction

Cancer survivorship care is defined as the care of a person with cancer from the time of diagnosis until the end of their life [1]. Quality cancer survivorship care includes prevention and surveillance for recurrences and new cancers; surveillance and management of physical and psychosocial effects; surveillance and management of chronic medical conditions; and health promotion and disease prevention, as well as care coordination and communication [2]. Cancer survivors face a variety of challenges across all of these domains. Unfortunately, their needs are often unmet, regardless of whether they receive care in oncology or primary care settings [3, 4], leading to the investigation of optimal models of care. To date, oncology-led survivorship care remains common, but its sustainability and effectiveness to comprehensively treat cancer survivors has been questioned [5]. One of the alternatives to oncology-led care is care led by the primary care physician (PCP). Traditional core values of primary care — including its comprehensive, patient-oriented, and continuous care — may render PCP-led care more fitting compared to oncologist-led care, though a recent overview of systematic reviews has not found consistent differences in the models [6].

While PCPs appear willing to provide care for cancer survivors [7], persistent barriers continue to hinder the provision of care, specifically a lack of perceived knowledge and expertise of PCPs regarding the necessary care [8]. Prior reviews have described the attitudes and perceptions of PCPs’ on cancer survivorship care provision, but did not specifically address the knowledge and confidence according to its domains [7, 9–11]. Thus, the purpose of this systematic review is to evaluate PCPs’ knowledge and confidence in general and categorized by the domains of cancer survivorship care. In turn, this will help to inform the role of the PCP in the provision of cancer survivorship care and development of future interventions.

## Methods

### Design

This systematic review was prepared, adhering to the Preferred Reporting Items for Systematic Review and Meta-Analysis Protocols (PRISMA-P) guidelines [12]. The review protocol was registered with PROSPERO (CRD42022333944) prior to review commencement.

### Main outcomes of interest

PCPs’ self-reported knowledge and confidence in providing cancer survivorship care were the main outcomes of interest. For the purpose of this review, knowledge was defined as the awareness, understanding, and skills obtained by experience or training, whereas confidence was defined as the feeling or belief to trust in oneself and one’s abilities, including self-efficacy, preparedness, and comfort in providing care. Studies that reported knowledge barriers and educational needs of PCPs were also included. We excluded studies if they only reported PCPs’ attitudes, beliefs, expectations, and preferences regarding the provision of cancer survivorship care.

### Search strategy

A medical librarian (FJ) performed the initial search for studies in PubMed using MeSH terms related to survivorship care, primary care physicians, and the main outcomes. The MeSH terms were translated into corresponding terms to search the following databases: Ovid MEDLINE, CINAHL, Embase, and PsycINFO. Databases were searched for studies dating from inception to 01 July 2022.

### Eligibility criteria

The population of interest was physicians providing primary or community-based survivorship care for cancer patients. The name for these physicians differs around the world and can include (but is not limited to): primary care physicians (PCPs), general practitioners (GPs) (sometimes referred to as GP specialists), and family physicians (FPs). Throughout this paper, “PCPs” will refer to all of these types of physicians. As the role of clinicians and nurses vary internationally, we focused this analysis on physicians only. While we included studies that surveyed different types of PCPs, we excluded those that did not report findings for PCPs alone. Any study design, including both quantitative and quantitative, were included. Letters to the editor and conference abstracts/papers that did not have a full manuscript available were not eligible. There were no restrictions based on type of cancer, publication date, or language. Studies were eligible if they described cancer survivorship care as the main topic or outcome of interest and reported on the main outcomes, described above.

### Screening and data abstraction

Title and abstract screening was performed by two authors (JV and BW) using Covidence software [13]. Subsequently, all authors performed full-text screening. Full-texts were assessed independently by two authors. All authors performed data abstraction. Each paper had one author assigned to extract data and one author to check for accuracy. The following data were extracted: characteristics of the included study (author, year, country, aim, and methods), description of PCPs (including age, sex, previous training/certification, work setting, and number of years in practice), cancer survivor population of interest (general, adolescents/young adults, childhood, and specific cancer type), and main outcomes. Quantitative data abstraction included relevant numerical data, while qualitative data abstraction included synthesized findings. Screening and data abstraction discrepancies were managed by the two authors until consensus was reached through discussion. A third author was included in the discussion if necessary.

### Quality appraisal

Quality appraisal assessments were performed using the Joanna Briggs Institute (JBI) Critical Appraisal tools for “Analytical Cross Sectional Studies” [14] and “Qualitative Research” [15]. Questions 3 and 4 from the “Analytical Cross Sectional Studies” checklist were not applicable to the main outcome and therefore not used. Similar to full-text screening, two authors independently conducted quality appraisal assessments of the eligible studies.

### Data synthesis

Characteristics from all studies were compiled and presented in tabular format. Quantitative outcomes were analyzed using the framework method and mapped to the different domains of cancer survivorship care as proposed by Nekhlyudov et al. [2] specifically focusing on (1) prevention and surveillance for recurrences and new cancers; surveillance and management of (2) physical effects and (3) psychosocial effects; (4) surveillance and management of chronic medical conditions, and (5) health promotion and disease prevention. The framework has been previously applied to systematic reviews assessing cancer survivorship care and educational programs [6, 16–18]. Qualitative and other related outcomes were reported narratively.

## Results

**Fig. 1:**
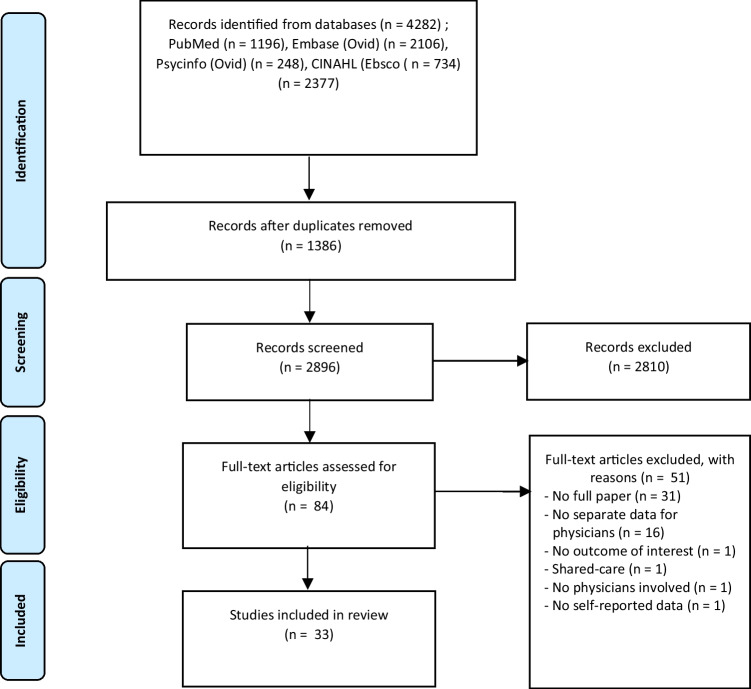
PRISMA flow diagram showing study selection process

Initial database searches resulted in 2896 potentially eligible records. After title, abstract, and full-text screening, 33 papers were included, representing 28 unique studies (Fig. 1). The papers by Bober et al. and Park et al. were based on the same dataset [19, 20]. Similarly, 5 other studies were based on the same dataset [21–25], but were assessed as individual studies as they generally reported different outcomes.

### Characteristics of the included studies

We included 22 quantitative studies (all cross-sectional survey design) [19–40], 8 qualitative studies (7 interviews [41–47] and 1 focus group [48]), and 3 mixed-methods studies [49–51] (Table 1). The quantitative data reported by Heins et al. was not within the scope of this review, so only the qualitative data for this study was extracted [50]. The papers originated from 8 countries, most of which in North America (*n* = 23) and Europe (*n* = 8). The cancer survivor population of interest was not specified (*n* = 11) or focused specifically on survivors of breast cancer (*n* = 4), prostate (*n* = 4) or other type of cancer (*n* = 4). Some papers focused on multiple types of cancer (*n* = 5), childhood cancer (*n* = 3), or cancer in adolescents/young adults (*n* = 2). For the survey studies, sample sizes ranged from 29 to 1124 PCPs, with varying degrees of participation (from 14.9 to 65.1%). Overall, the surveyed population was predominantly male, White, around 50 years of age, and working in suburban or urban areas. All quantitative studies used Likert scale questions to gauge knowledge and confidence of PCPs. Likert scales ranged from 3 to 7 points, and outcomes were reported in different formats (%, means and percentiles).

**Table 1:** Characteristics of the included studies

Author, year, country	Aim	Cancer survivor population of interest	Methods	Population (*N*), sample (*n*), response (%)*	Primary care physician description	Knowledge and confidence outcomes	Other related outcomes
*Quantitative*							
Berry-Stoelzle et al. 2019, USA [26].	To identify PCPs’ needs for care of a patient who has had cancer.	Not further specified.	Survey among Iowa family physicians in Nov 2017.	*N* = 274, *n* = 82, 30%.	Male (62%), mean age: 57 (range 32–84), living in metropolitan areas (45%).	*Confidence in managing mental health and side effects of cancer therapies (4-point Likert questions).	NA.
Bober et al. 2009 [19], Park et al. 2009, USA [20].†	To understand challenges in survivorship care and to investigate how factors may be associated with the PCPs’ attitudes and approaches to survivorship care.	Not further specified.	Survey among community-based and academic-based internists in Denver, Colorado, between Sep 2007 and March 2008.	*N* = 319, *n* = 227, 71%.	Male (59%) and Caucasian (85%). Board certified in Internal Medicine (86%). Average years in practice: 21.2.	*Confidence/*preparedness to manage long-term effects of cancer treatment (4-point Likert questions). *Confidence/*comfort in providing care (4-point Likert question).	Barriers to care (4-point Likert questions).
Chou et al. 2020, USA [27].	To understand physicians’ comfort prescribing medications for comorbidities in patients with cancer.	Adults with cancer, not further specified.	Survey among healthcare practitioners with a MD or DO degree in May 2017.	*N* = 3.518, *n* = 50.	Male (78%), mean age: 54, White (60%), working in suburban setting (62%). Mean years in practice: 23.	*Confidence/*comfort-level self-prescribing 6 classes of medications frequently prescribed to cancer patients to manage cardiometabolic and psychiatric conditions (7-point Likert questions).	NA.
Chow et al. 2017, Canada [28].	To determine the educational needs of PCPs in the management of chronic pain and to gauge their interest in fulfilling this role in the cancer continuum of care.	Non-palliative cancer patients who completed treatment.	Survey among PCPs between June and August 2016.	*N* = 12.000, *n* = 162.	Physicians (85.2%) working in communities with populations of 10.000–100.000 (41.9%) and practicing in a group practice/family health team (59.2%). Average years in practice: 24.	*Knowledge about the management of chronic pain related to cancer treatment (4-point Likert question).	Barriers to care (1 multiple choice question), and educational needs (1 multiple choice question).
Fidjeland et al. 2015, Norway [29].	To investigate GPs’ experiences in the provision of follow-up care for cancer patients, with an emphasis on collaboration with secondary care.	Patients with gynecological cancer.	Survey among GPs in seven regions between Jan 2013.	*N* = 1457, *n* = 317.	Male (60%), age 41–60 (51%), working in an urban setting (87%). Specialist in general medicine (73%).	*Knowledge/*skills to provide follow-up for cancer patients (3-point Likert question). *Confidence to take over responsibility for follow-up care (3-point Likert question).	NA.
González Carnero et al. 2013, Spain [30].	To identify the current training needs in the care of patients with cancer of family doctors and their attitude towards it.	Patients diagnosed with cancer, terminal patients, and survivors	Survey among family doctors in Castilla La Mancha in 2010.	*N* = 1154, *n* = 172, 14.9%.	Male (51.2%), mean age: 49.1, Tutor of residents (44.2%), working in rural areas (44.2%).	*Confidence to treat oncological patients (3-point Likert question).	Educational needs (6-point Likert questions), and barriers to care (lack of involvement).
Mani et al. 2020, USA [31].	To assess perceived barriers to delivery of care for cancer survivors, resources available to care for cancer survivors, practices for care coordination with hematologist-oncologists, and preferred models of delivery.	Adult hematologic cancer and hematopoietic cell transplantation survivors.	Survey among PCPs at two large integrated healthcare systems (Cleveland Clinic and Mayo Clinic). Date study conducted not reported.	*N* = 302, *n* = 86, 29%.	Trained in: internal medicine (70%), family medicine (24%), pediatrics (6%). Tutor of students/residents (79%). Median years since graduation: 17.	*Confidence to provide care to (3-point Likert questions); (1) a 40-year-old female, 3 years after completion of therapy for acute myeloid leukemia, (2) an identical patient that had also received an allogeneic HLA-identical sibling donor transplant.	Barriers to care (4-point Likert questions).
McDonough et al. 2019, USA [32].	To evaluate PCP experiences and perspectives in cancer survivorship and to identify practical opportunities to improve care.	Not further specified.	Survey among PCPs in practices affiliated with an academic medical center (Massachusetts General Hospital) in Jan–Feb 2018.	*N* = 225, *n* = 117, 52%.	Female (68%), median age: 53 (range 30–71), working in practice for >10 years (82%).	*Confidence/*preparedness for cancer care provisions (4-point Likert questions).	Barriers to care (3-point Likert questions).
Nathan et al. 2013, USA and Canada [33].	To determine family physicians’ comfort with caring for childhood cancer survivors (CCS).	Adolescent and young adult (AYA) cancer survivors - defined as patients diagnosed with cancer at or prior to 21 years of age who are alive at least 5 years from initial diagnosis	Survey among US family physicians (from the American Academy of Family Physicians) and Canadian participants (family physicians and general practitioners) between Dec 2010 and Oct 2011.	*N* = 2520, *n* = 1124, 45%.	Male (62%), median age: 53 (IQR 43–60), median years in practice: 22 (IQR 11–30), working in a group practice (39%).	*Knowledge/*familiarity of available surveillance guidelines (7-point Likert-style question). *Confidence/*comfort with caring for AYA survivors of specific cancers (acute lymphoblastic leukemia, Hodgkin lymphoma, andosteosarcoma) (7-point Likert-style questions).	NA.
Potosky et al. 2011 [21], Cheung et al. 2013 [22], Klabunde et al. 2013 [23], Nekhlyudov et al. 2014 [24], Virgo et al. 2013, USA [25].	To describe and compare PCPs’ and oncologists’: 1) preferred model of follow-up care; 2) perceptions of PCPs’ skills in providing follow-up care; 3) confidence in knowledge of components of follow-up care; and 4) cancer surveillance practices.	Breast and colon cancer survivors.	Nationwide survey among PCPs and oncologists between March and December 2009.	*N* = 3.596 (includes both PCPs and oncologists), *n* = 1062 (PCPs only), weighted response 65.1%.	Male (65%), age 40–49 (33%), White (70%). Trained in: family medicine (43 %), general internal medicine (37%), ob/gyn (20%). Working in an office practice (87.2%).	*Knowledge/*skills to initiate screening and diagnostic evaluations to detect recurrent cancer, to care for late physical effects, and to provide psychosocial support (5-point Likert questions). *Confidence to detect recurrent disease, to care for late physical effects, and to provide psychosocial support (3-point Likert questions).	Barriers to care.
Radhakrishnan et al. 2020, USA [34].	To describe PCP involvement in thyroid cancer long-term surveillance, and their confidence in handling various aspects of thyroid cancer survivorship care.	Thyroid cancer survivors.	Survey among PCPs from the Georgia and LA SEER registries between Aug 2018 and Aug 2019.	*N* = 289, *n* = 162, 56%.	Male (52.9%), White (67.3%), working in private practice (74.1%), practicing for >20 years (63.2%).	*Knowledge/*skills to initiate appropriate screening to detect recurrent thyroid cancer (5-point Likert question). *Confidence in discussing follow-up care, including the role of random thyroglobulin levels in surveillance, role of neck ultrasound, when to end surveillance, and when to refer back to the specialist (5-point Likert questions).	NA.
Roorda et al. 2013, Netherlands [35].	To explore (a) the discharge of breast cancer patients to primary care, at the end of hospital follow-up and (b) the experiences and views of GPs regarding transfer of follow-up.	Breast cancer survivors.	Survey among GPs in three Northern provinces between Sep and Oct 2010.	*N* = 949, *n* = 502, 53%.	Male (66.3%), >44 years of age (71%), practicing >10 years (74%), working in a group/health center (36%).	*Knowledge/*skills to take over breast cancer follow-up and examine irradiated breasts to detect local recurrences and second tumors (3-point Likert questions).	Recommendations for providing survivorship care.
Sima et al. 2014, USA [36].	To identify facilitators and barriers to PCPs providing optimal care for CCS.	CCS.	Survey among PCPs from general internal medicine and family practice between Feb–Apr 2008.	*N* = 1.500, *n* = 351, 23%.	Male (60.1%), Trained in: family practice (64%), internal medicine (36%). Graduated between 1990 and 2000 (38.7%). Main practice setting: office-based (76.7%).	*Knowledge/*perception that medical training was adequate to recognize late effects of chemotherapy, cancer surgery and radiation (5-point Likert questions). *Knowledge/*awareness of practice guidelines for CCS yes vs. no question).	NA.
Skolarus et al. 2013,USA [37].	To better understand PCPs’ beliefs and practice patterns with regard to prostate cancer survivorship.	Prostate cancer survivors.	Survey among PCPs (and nurse practitioners and physician assistants) licensed to practice in the state of Michigan in 2006.	*N* = 5.687 (including NPs and Pas), *n* = 670, 15.7%.	Male (53.7%), median age: 50 (range 24–88), White/Caucasian (83.8%), 21–30 years since graduation (29.6%), working in a single specialty group practice (36.7%).	*Confidence/*comfort level with treating survivors’ incontinence, impotence, bowel problems and psychosocial concerns (3-point Likert questions).	NA.
Smith et al. 2011, Canada [38].	To assess the confidence of PCPs in their ability to care for survivors of breast cancer, and to explore ways to improve their success in providing such care.	Women with non-metastatic breast cancer.	Survey among PCPs (from the British Columbia Cancer Agency) between June 2007 and Aug 2008.	*N* = 997, *n* = 587, 59%.	Responses were anonymous. 61% of respondents had more than 10 survivors in their practice.	*Confidence in managing aspects of breast cancer follow-up (such as screening for recurrence, adjuvant hormone therapy, etc.) (3-point Likert questions).	Educational needs (free-text question).
Suh et al. 2014, USA [39].	To determine general internists’ self-reported attitudes and knowledge about the care of CCS.	CCS (diagnosed ≤21 years) at least 5 years from treatment completion.	Nationwide survey among general internists between Sep 2011 and Aug 2012.	*N* = 1801, *n* = 1110, 61.6%.	Male (60.7%), mean age: 44 (SD 10), mean years in practice: 11.9 (SD 9.7), working in a group practice of ≥3 physicians (27.6%).	*Knowledge/*familiarity with the available monitoring guidelines for childhood, adolescent, and young adult cancer survivors (7-point Likert questions). *Confidence/*comfort level with caring for survivors of acute lymphoblastic leukemia, Hodgkin lymphoma, and osteosarcoma (7-point Likert questions).	NA.
Walter et al. 2015, UK [40].	To examine the current practices and views of GPs in England on providing care for those living with, and beyond, cancer.	People living with, and beyond, cancer (excluding non-melanoma skin cancer) or those who have completed definitive primary cancer treatment.	Nationwide survey among GPs in June 2014.	*N* = unclear (at the time of recruitment the panel size was approximately 10.000), *n* = 500.	Male (75%), >15 years of experience (73%), GP partner (79%), working in an urban setting (54%). Previous training in general care for cancer (50%).	*Knowledge/awareness of possible late cardiovascular and bone effects following treatment (5-point Likert questions). *Confidence in their role of providing specific care for cancer survivors (5-point Likert question).	Educational needs.
*Qualitative*							
Duffey-Lind et al. 2006, USA [41].	To identify and describe the experiences of adolescent cancer survivors and that of their parents and pediatricians from the completion of therapy to the first few years off of therapy.	Adolescent survivors (14–18 years of age) that were 1–5 years off treatment.	Semi-structured interviews with community-based pediatricians (and adolescent cancer survivors and their parents). Date interviews conducted not reported.	*N* = 7, *n* = 3, 43%.	Baseline characteristics not reported.	NA.	The interview guide included questions regarding (1) specific cancer-related information that was available to physicians and (2) what information they gave to their patients.
Fox et al. 2021, Australia [42].	To explore the perspectives of the roles of general practice team members in the delivery of cancer survivorship care.	Not further specified.	Semi-structured interviews with GPs (and practice nurses and practice managers) between Feb and Aug 2020.	*N* = unclear, *n* = 10.	Male (*n* = 5), age 31–40 (*n* = 6), working in a metropolitan area (*n* = 10). Years of experience: 6–10 (*n* = 4).	NA.	An interview guide explored the roles of GPs. Interviews were flexible and iterative, and framed as a conversation.
Margariti et al. 2020, UK [43].	To examine the preparedness, concerns and experiences of GPs in relation to their role in providing follow-up care to prostate cancer survivors.	Prostate cancer survivors.	Semi-structured interviews with GPs between May–Sep 2015.	*N* = 78, *n* = 20.	Male (*n* = 10), mean years of experience: 19.1. Special interest or qualification in prostate cancer (*n* = 5).	Topics included current experience with prostate cancer survivors, preparedness and willing-ness to take over their follow-up care and challenges encountered.	NA.
Radhakrishnan et al. 2019, USA [44].	To explore prostate cancer survivorship care among PCPs and cancer specialists within an integrated healthcare delivery system.	Prostate cancer survivors.	Semi-structured interviews with PCPs (and cancer specialists) from the Veterans Health Administration. Date interviews conducted not reported.	*N* = unclear, *n* = 4.	Baseline characteristics not reported.	An interview guide was developed based on theoretical domains framework, which consists of 14 domains, including knowledge, skills and beliefs about capabilities.	NA.
Sarfo et al. 2022, Netherlands [48].	To explore views of GPs and occupational physicians (OPs) on the role of GPs in work guidance of cancer patients.	Not further specified.	Focus group interviews with GPs (and OPs) between 2016 and 2019.	*N* = unclear, *n*= 17.	Male (*n* = 8), mean age: 53.1 (SD 7.4), mean years of experience: 20.5 (SD 7.7), working in a metropolitan area (76.5%).	NA.	An interview guide explored the perspectives of GPs regarding their role in the work guidance of cancer patients.
Signorelli et al. 2019, Australia and New Zealand [45].	To explore the feasibility of PCP-led survivorship care from survivors’/parents’ and their PCPs’ perspectives through a two-stage study.	CCS.	Semi-structured interviews with PCPs (and young survivors and their parents). Date interviews conducted unknown.	*N* = 160, *n* = 74 (data saturation after *n* = 51), 46%.	Male (57%), working in major cities (65%), average years of experience: 28.3 (SD 11.7).	The interview guide asked about PCP confidence in delivering survivorship care and understanding survivors’ current and future follow-up needs.	NA.
Thamm et al. 2022, Australia [46].	To understand GP’s perspectives on their role in addressing financial toxicity (FT) amongst cancer patients in the primary care setting.	Not further specified.	Semi-structured interviews with GPs between Nov 2019 and June 2020.	*N* = unclear, *n* = 20.	10–20 years of experience (*n* = 5), working in metropolitan areas (*n* = 13).	NA.	An interview guide was developed to understand GP’s role in addressing FT.
Vos et al. 2022, Netherlands [47].	To explore GPs’ experiences with the delivery of a colon cancer survivorship care intervention, and study its implementation into clinical practice.	Colon cancer survivors.	Semi-structured interviews were held parallel to a RCT on GP-led follow-up. Interviews conducted after 1- and 5-year of follow-up.	*N* = 27, *n* = 17.	Male (*n* = 7), mean age of: 46, self-employed (*n* = 12), median years of experience: 13.	An interview guide was based on Normalisation Process Theory which includes skills and training as part of the collective action construct.	NA.
*Mixed-methods*							
Dawes et al. 2015, USA [49].	To assess PCPs’ knowledge of, attitudes toward, and confidence in providing survivorship care to patients with breast cancer.	Breast cancer survivors.	Survey among PCPs (and non-physicians) from two primary care networks within a public safety-net system. Date study conducted not reported.	*N* = 115, *n* = 42 (*n* = 59 including non-physicians), 51%.	Respondents (including non-physicians) were trained in: internal medicine (56%), family medicine (36%), ob/gyn (8%). Training in survivorship (75%).	*Knowledge/*skills to provide follow-up care and initiate diagnostic or screening workup for recurrence (4-point Likert questions). *Confidence in providing survivorship care, including cancer surveillance, and addressing physical and psychosocial comorbidities (3-point Likert questions).	All providers were invited to attend focus group sessions to expand upon their experiences and concerns regarding survivorship care. These results were not stratified for (non-)physicians.
Heins et al. 2017, Netherlands [50].	To assess acceptability and feasibility of GP-led follow-up for prostate cancer in a pilot study.	Older prostate cancer survivors (≥ 65 years) with at least one comorbidity.	A mixed-methods feasibility pilot study, which included semi-structured interviews with GPs (and urologists).	*N* = unclear, *n* = 14.	Not reported.	NA (quantitative data is not within the scope of this review).	Researchers asked predefined questions regarding satisfaction, logistical and/or practical problems, uncertainties, and communication.
Stephens et al. 2021, USA [51].	To examine PCPs’ internal (confidence, training) and external factors (communication, receipt of survivorship care plans) regarding their provision of survivorship care to older breast cancer survivors.	Older (≥65 years) breast cancer survivors.	A mixed-methods study using a survey and semi-structured interviews among PCPs affiliated with the Ohio State University. Date study conducted not reported.	*N* = 100, *n* = 29 (of which 10 participated in semi-structured interviews).	Male (31.1%), mean age: 43.5 (SD 11.2), White (71.4%), mean years in practice: 13.5 (SD 12.4). Trained in: family medicine (51.7%), internal medicine (48.3%).	*Confidence in addressing late effects of treatment and comorbidities (5-point Likert questions).	In interviews, PCPs were asked to share their comments about addressing survivorship issues and outcomes and ideas for improving care.

*PCP* primary care physician, *GP* general practitioner, *CCS* childhood cancer survivor, *AYA* adolescent and young adult, *SD* standard deviation, *OP* occupational physicians, *FT* financial toxicity

^*^Population size refers to the total number of PCPs eligible. Sample size is the number of PCPs included in the study. Response rate in % if provided

^†^Characteristics reported in the first article are shown

### Quality of the evidence

**Fig. 2:**
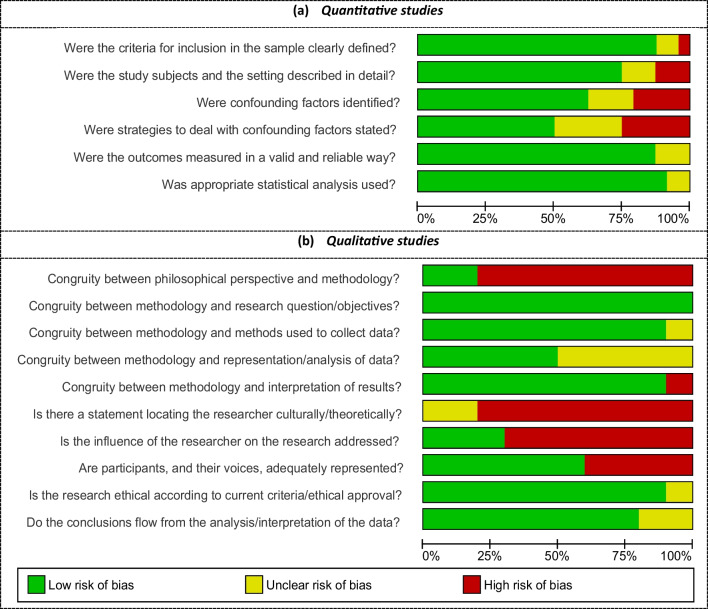
Quality appraisal of the included papers. **a** Assessment of quantitative studies using the Joanna Briggs Institute (JBI) Critical Appraisal tools for “Analytical Cross Sectional Studies”[14]. **b** Assessment of qualitative studies using the JBI tool for “Qualitative Research”[15]

For the quantitative studies, risk of bias was often related to the identification of possible confounding factors and strategies to deal with them (*n* = 12) [19–23, 28–30, 35, 37, 40, 51] (Fig. 2). Some studies did not provide the time period of the survey (*n* = 5) [19, 24, 31, 49, 51] or provided little information about the population and sample sizes (*n* = 2) [28, 49]. In three studies, there was limited information about the surveyed population in general [28, 30, 32]. For the qualitative studies, none provided a clear statement locating the researchers’ cultural or theoretical background. Suboptimal quality was also related to a lack of underlying theoretical premises (*n* = 8) [41, 43–45, 47, 48, 50, 51], and addressing the influence of the researcher on the research (*n* = 7) [41, 43–45, 48, 50, 51]. Ratings of each individual study are provided in Supplementary file 1.

## Quantitative outcomes

**Fig. 3:**
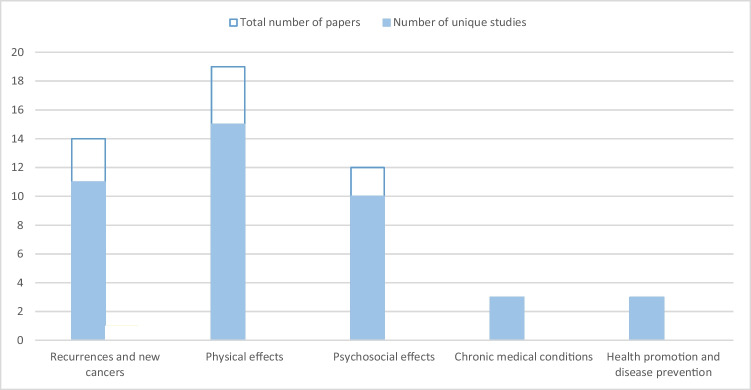
The number of papers included in the review grouped by the outcomes on the cancer survivorship care domains. *Because some of the papers were based on the same dataset, both the total number of papers, and the number of unique studies is provided

All outcomes were mapped to the different survivorship care domains and can be found in Supplementary file 2. Most papers described knowledge and confidence in the management of physical (*n* = 19) and psychosocial effects (*n* = 12), and of prevention and surveillance for recurrences and new cancers (*n* = 14) (Fig. 3). Outcomes of some studies (*n* = 5) could be mapped to multiple domains [19, 20, 25, 30, 33].

### Prevention and surveillance for recurrences and new cancers

Ten studies described the PCPs knowledge, mainly described as skills, to conduct routine follow-up and management of recurrences and new cancers [21–23, 29, 33–36, 39, 49]. Five studies assessed the skills to provide “routine follow-up cancer care” (*n* = 5) [21, 22, 29, 35, 49]. In four of these studies, 41–59% of PCPs agreed that they had the necessary skills to provide follow-up [21, 22, 35, 49]. In a Norwegian study of patients with gynecological cancer, up to 78% of PCPs agreed or partly agreed they had the necessary skills to provide follow-up cancer care [29]. In other studies, between 62 and 78% of PCPs “somewhat or strongly agreed” they had the necessary skills to initiate appropriate screening and detection of recurrences (*n* = 5) [21–23, 34, 49]. In a single study from the Netherlands, only 20% agreed they “had the skills necessary to examine irradiated breasts to detect local recurrences and second tumors” [35]. Three studies also described the awareness and familiarity of surveillance guidelines [33, 36, 39]. In two of these studies, only 9–12% of PCPs felt at least “somewhat familiar” (Likert score ≥5) with guidelines [33, 39]. Lack of knowledge of evidence-based guidelines was mentioned as a barrier to providing care in two different studies [28, 31].

Confidence to screen for cancer recurrence was rated much lower across all the included studies. Between 6-42% felt very confident or prepared to do so (*n* = 6) [21, 29, 31, 32, 34, 49].

### Surveillance and management of physical effects

Knowledge and confidence in management of physical effects was described in 13 studies. About half of these studies (*n* = 7) did not specify the physical effect under evaluation [21, 22, 24, 32, 39, 49, 51], while others (*n* = 6) focused on specific symptoms, such as fatigue and treatment-related osteoporosis [26, 28, 36–38, 40]. Only four studies examined knowledge of PCPs regarding physical effects [24, 28, 36, 40]. In three of these studies, 30–32% of PCPs reported "somewhat or good” knowledge of physical effects [24, 28, 36]. Overall, between 10-37% of PCPs reported (high) confidence in providing care for physical effects (*n* = 8) [21, 22, 32, 37, 39, 40, 49, 51]. Confidence was rated differently for specific physical effects [26, 38], for example 79% of PCPs felt confident in managing fatigue, whereas only 16% felt confident in managing chemobrain [26].

### Surveillance and management of psychosocial effects

None of the included studies measured knowledge of psychosocial effects. Seven studies examined PCPs’ confidence in the management of psychosocial effects [21, 26, 32, 37, 40, 49]. In general, a greater proportion of PCPs were confident in managing psychosocial effects than physical effects. Between 24 and 47% of PCPs felt very confident or prepared to manage psychosocial symptoms and adverse effects [21, 32, 37, 40, 49]. In the study by Berry-Stoelzle et al., in which a 4-point Likert scale was reduced into “confident” vs. “not confident”, almost 100% of PCPs were confident in managing depression, anxiety and sleep disturbances [26]. In a different study, PCPs reported overall “good/adequate” confidence in managing anxiety or fear of recurrence (0.97 on a scale of 1.0) [38]. Notably, one study reported high confidence in managing psychological symptoms (47%), but low confidence in providing advice concerning work and/or finances (19%) [40].

### Surveillance and management of chronic medical conditions

Three studies examined confidence and comfort in managing chronic medical conditions [27, 31, 51]. None reported knowledge. One study showed high comfort in prescribing medications for cardiometabolic and psychiatric comorbidities in patients with cancer [27]. Another study showed high confidence in managing general medical issues (77%), but much lower confidence in managing cancer-related medical issues (13%) [31]. In the study by Stephens et al., 41% felt very confident to address chronic comorbidities [51].

### Health Promotion and Disease Prevention

Three studies investigated health promotion and disease prevention [32, 38, 40]. None reported knowledge. Between 51 and 57% of PCPs felt very prepared to provide routine age-appropriate preventive care and vaccinations [32]. In two different studies, PCPs reported good confidence in providing lifestyle recommendations [38, 40].

### Knowledge barriers and educational needs

Several quantitative studies highlighted the need for education and training on survivorship care issues (*n* = 9) [19, 20, 25, 28, 30–32, 35, 40]. These other outcomes can be found in Supplementary file 3. Inadequate or lack of formal training was reported by up to 72% of PCPs (*n* = 5) [19, 20, 25, 30, 32]. Three studies also described the educational needs of PCPs [28, 30, 40]. In the study by Walter et al., there was a great desire for education on physical effects following cancer treatment (76–86%), but to a lesser extent on psychosocial effects (36-52%) [40].

## Qualitative outcomes

Of the included qualitative studies, some specifically aimed to describe knowledge and confidence in care (*n* = 5) [43–45, 47, 51], while others described it secondary to their aim (*n* = 5) [41, 42, 46, 48, 49]. Many studies described a lack of knowledge to provide survivorship care (*n* = 9) [41, 43–49, 51]. PCPs felt that they need to be educated regarding the follow-up plan/surveillance guidelines, including the interpretation of follow-up test results (*n* = 4) [41, 43, 44, 49], but also potential late effects of therapy (*n* = 3) [41, 47, 51]. Three studies mentioned insufficient knowledge of PCPs in work regulations and addressing financial burden of cancer patients [40, 46, 48]. Two different studies showed that lack of knowledge, and therefore lower confidence levels, depended on the cancer survivor population of interest, in this case childhood cancer survivors [45] and older breast cancer patients (≥65 years) [51]. Some PCPs felt well-prepared to provide survivorship care and subsequently confident to do so [42–44, 47, 50]. One study indicated that this confidence was grounded in the “wide experience of managing different cancers and chronic conditions other than cancer” [43]. All qualitative outcomes can be found in Supplementary file 4.

## Discussion

Our systematic review found 33 studies on self-reported knowledge and confidence of PCPs in providing cancer survivorship care, of which most focused on the management of physical (*n* = 19) and psychosocial effects (*n* = 12), and the prevention and surveillance for recurrences and new cancers (*n* = 14). Overall, a greater proportion of PCPs were confident in managing psychosocial effects after cancer (24–47%) [21, 32, 37, 40, 49] than physical effects (10–37%) [21, 22, 32, 37, 39, 40, 49, 51]. More PCPs reported having the knowledge, specifically skills, in initiating screening and detection of recurrences (62–78%) [21–23, 34, 49], than in providing routine follow-up care for cancer survivors (41-59%) [21, 22, 35, 49]. Even fewer PCPs felt very confident, or prepared, to provide routine follow-up care and detect recurrences (6–42%) [21, 29, 31, 32, 34, 49]. Knowledge of cancer surveillance guidelines, and the need to be educated on it, was mentioned in multiple studies [28, 31, 33, 36, 39, 41, 43, 44, 49]. While prior reviews addressed attitudes and preferences to provide cancer survivorship care [7, 9–11], to our knowledge, this is the first systematic review to specifically delineate PCPs’ knowledge and confidence in cancer survivorship care. Lack of knowledge and expertise of PCPs is mentioned in all reviews as a barrier to provide survivorship care for cancer patients [7, 9–11]. However, none of these previous reviews have focused specifically on the PCPs’ knowledge and confidence to provide survivorship care. A recent scoping review by Hayes et al. showed that the perceived lack of knowledge is more prevalent among PCPs, than specialists or cancer survivors [8]. Even though PCPs generally reported good or adequate skills in detecting recurrences [21–23, 34, 49] and providing follow-up care [21, 22, 35, 49], their confidence to do so was rated much lower than their knowledge [21, 29, 31, 32, 34, 49]. While our study did not address these factors, others have found lower confidence levels related to fewer (years of) experience [26, 31–34, 38, 39] and female sex [26, 30, 33, 39]. Confidence may also be related to the cancer survivor population of interest [29, 45, 51] as PCPs may only see a limited number of patients with particular types of cancer.

Previous reviews have indicated the need for additional education and training of PCPs on survivorship care issues [7–11]. A previous review by Chan et al. showed that survivorship education programs help increase knowledge and confidence [16] and assessed the domains of care that were included in such programs. Interestingly, the review found that educational programs tended to focus on physical and psychological effects, as well as cancer recurrence. These findings suggest that despite the attention being placed on these areas of cancer survivorship care, gaps in knowledge and confidence remain. Our study may help to inform future educational programs for PCPs, specifically focusing on managing the physical effects and prevention and surveillance for recurrences. Some studies highlighted the need for PCP education on addressing work and/or financial consequences of cancer treatment for patients [40, 46, 48]. While it is important for PCPs to understand these issues, it is not clear to what extent they feel willing and/or capable of addressing such concerns. We also found a lack of studies addressing knowledge and confidence in the management of chronic medical conditions and health promotion and disease prevention in cancer survivors. It is possible that when conducting research in this area, investigators have chosen to focus on cancer-specific domains rather than those that are already mainly addressed in primary care. However, primary care, with its holistic approach, has an important role in addressing these domains [52–54]. Focus on these domains in a PCP-led model could potentially improve outcomes of cancer survivors [53], particularly those who are older and have chronic medical conditions. It is important to emphasize that the educational programs that tackle the gaps in PCPs knowledge and confidence incorporate adult learning theory principles, including behavior change, in order to promote more tangible and sustainable changes in clinical practice [16]. In addition to educational programs, our study adds insights into the potential role of the PCPs in cancer survivorship care and the need for focused interventions in clinical practice. While PCPs appear willing to provide survivorship care [7], divergent views exist regarding the potential role of the PCP. Even though most PCPs believe that cancer survivorship care is within their purview, others consider follow-up after cancer the responsibility of the oncology specialist [11, 55]. These views are likely to depend on the context and setting in which survivorship care takes place. Most papers in our analyses originated from the USA in which survivorship care still relies mainly on oncology specialists’ expertise, despite ongoing efforts to bring survivorship care to the forefront of primary care [56]. In other countries, such as Canada, PCP-led care is more common and widely accepted [57]. This illustrates that further integration of survivorship care in primary care is possible. While we did not specifically address communication and coordination of care with oncology providers in our study, these factors are among the frequently reported barriers to the provision of quality care [7–11]. Interventions targeting communication regarding management of physical effects and surveillance for recurrences, using electronic health records or survivorship care plans, for example, may enhance PCPs knowledge and confidence.

We acknowledge several limitations of this study. First, most papers originated from the USA (*n* = 20), which may limit the generalizability of the results to other countries with different healthcare systems. Second, as described earlier, PCPs’ involvement, education and experience with cancer survivorship care differ around the globe. This is likely to affect PCPs’ self-reported knowledge and confidence to provide such care. Participation rates of the surveyed populations varied greatly (14.9 to 65.1%) which could further have an impact on the generalizability. PCPs working in rural areas were underrepresented in the included studies but are likely to have different experience in providing care for cancer survivors than those working in (sub)urban areas. Third, most of the included studies had multiple aims, and measuring knowledge and confidence was often just a small part of those aims. While we focused on knowledge and confidence, maintaining to strict study definitions, the terminology used by the included studies varied. Studies more often focused on confidence in care, than knowledge per se. Future studies should therefore include both outcomes, to help us understand how these two interrelate. It is also important to apply universally-agreed upon terminology to make sure that these studies measure the same outcomes and are consistent. This will permit comparison across populations and interventions [58]. Furthermore, because we specifically focused on physicians’ self-reported data, we excluded 16 studies that did not report these data separately from the other members of the primary care team, such as advance practice clinicians and nurses (Fig. 1), who play an important role in caring for cancer survivors. However, these excluded papers still contain relevant information, and likely provide further information on the knowledge and confidence of other primary care team members. Finally, of the 33 papers, 7 used data from the same 2 studies. Even though the sample sizes and reported outcomes differed in the follow-up papers of the same datasets [19–25], there is likely overlap. However, as we are not conducting meta-analyses, we do not believe that this has important implications on our findings. Despite the limitations, our study adds to the existing literature by including quantitative and qualitative studies, and by characterizing the outcomes by cancer care domains, using previously applied methodology [6, 16–18].

In summary, our study found that PCPs’ self-reported knowledge and confidence in cancer survivorship care varies across the care domains and is specifically limited in management of physical effects and prevention/surveillance for recurrences and new cancers. These results provide insights into the potential role of PCPs in cancer survivorship care, and the development of future educational programs medical education and targeted interventions.

## Supplementary information

ESM 1 (DOCX 108 kb)

## Data Availability

All data abstracted and analyzed during the review process are included in the article and its supplementary files.
