# Supplementary material for: Primary care physicians’ knowledge and confidence in providing cancer survivorship care: a systematic review
Source: J Cancer Surviv. 2023 May 12;18(5):1557–73. doi: 10.1007/s11764-023-01397-y (PMC11424677; doi:10.1007/s11764-023-01397-y)
Supplement: Supplementary file 1 — (DOCX 108 kb) [file 11764_2023_1397_MOESM1_ESM.docx]

**Supplementary file 1 – Quality appraisal of each individual study.**

**Figure 1 Assessment of quantitative studies using the Joanna Briggs Institute (JBI) Critical Appraisal tools for “Analytical Cross Sectional Studies^(1)^.**

******

**Figure 2 Assessment of qualitative studies using the Joanna Briggs Institute (JBI) tool for “Qualitative Research”^(2)^.**

**Supplementary file 2 – Quantitative outcomes according to the cancer survivorship care domains.**

| **Author, year, country** | **Prevention and surveillance for recurrences and new cancers** | **Surveillance and management of physical effects** | **Surveillance and management of psychosocial effects** | **Surveillance and management of chronic medical conditions** | **Health promotion and disease prevention** |
| --- | --- | --- | --- | --- | --- |
| Berry-Stoelzle et al., 2019, USA^(3)^. |  | Not confident vs. confident in managing: chemobrain (84% vs 16%, n = 74), fatigue (21% vs 79%, n = 75), pain (7% vs 93%, n = 75), cardiotoxicity (71% vs 29%, n = 75), skin changes (35% vs 65%, n = 75), musculoskeletal disturbances (20% vs 80%, n = 74), lymphedema (32% vs 68%, n = 75), sexual dysfunction (33% vs 67%, n = 75), bowel or bladder incontinence (23% vs 76%, n = 75), early-onset menopause (20% vs 80%, n = 75). | Not confident vs. confident in managing: depression (0% vs 100%, n = 78), anxiety (0% vs 100%, n = 78), alcohol use (18% vs 82%, n = 77), sleep disturbances (1% vs 99%, n = 78), other substance abuse (38% vs 62%, n = 77). |  |  |
| Bober et al, 2009, USA^(4)^. |  | Unprepared vs. prepared to evaluate long-term effects (49.8% vs. 52.1%) and manage long-term effects (47.9% vs. 52.1%) (n = 215).* | “…” |  |  |
| Cheung et al, 2013, USA^(5)^. | PCPs have the skills (somewhat/strongly agree) to provide routine cancer follow-up: (57%, n = 558), to detect and work-up recurrent cancers (74%, n = 720)  Very confident in surveillance testing to detect recurrent cancer (34%, n = 349). | Very confident in managing long-term and late physical adverse effects (19%, n = 196). |  |  |  |
| Chou et al, 2020, USA^(6)^. |  |  |  | Great comfort initiating cardiometabolic medications (mean comfort-level scale score (SD) = 96.8% (5.56%). PCPs were comfortable initiating each of the 3 cardiometabolic medication classes (mean on a 7-point scale (SD): antihypertensives = 6.80 (0.404); antidiabetics = 6.78 (0.418); antihyperlipidemics = 6.74 (0.443)).  PCPs were comfortable initiating psychiatric medications (mean psychiatric condition comfort-level scale score (SD) = 85.7% (12.51%)). PCPs were also comfortable initiating each of the 3 psychiatric medication classes (mean on a 7-point scale (SD): antidepressants = 6.56 (0.577); anxiolytics/sedatives/hypnotics = 6.18 (1.004); antipsychotics = 5.26 (1.651)). |  |
| Chow et al, 2017, Canada^(7)^. |  | Limited knowledge of cancer treatment-related pain (n = 14, 9.5%), some knowledge (n = 148, 52.7%), good knowledge (n = 47, 31.8%). |  |  |  |
| Dawes et al, 2015, USA^(8)^. | PCPs have the skills (somewhat/strongly agree) to provide follow-up care related to cancer or cancer treatment (n = 18, 43%), to initiate diagnostic or screening workup for recurrence (n = 26, 62%).  Very confident to provide appropriate surveillance to detect disease recurrence (n = 9, 21%). | Very confident to address late-term physical adverse effects (n = 6, 14%). | Very confident to identify potential psychosocial adverse effects (n = 10, 24%). |  |  |
| Fidjeland et al, 2015, Norway^(9)^. | 78% agreed or partly agreed that GPs have the necessary knowledge/*skills to provide follow-up cancer care.  42% would feel confident to take over responsibility for follow-up within three years after active treatment, yet approximately 10% reported ''never''. 69% who responded ‘‘never’’ argued that it was safer if a gynecologists' assumed responsibility. |  |  |  |  |
| González Carnero et al, 2013, Spain^(10)^. |  | Low confidence in treating oncological patients (4.7%), medium (74.4%) and high (20.9%).* | “…” |  |  |
| Klabunde et al, 2013, USA^(11)^. | 63% agrees that PCPs have the skills to provide follow-up care for cancer or its treatment effect, while 78% agrees that PCPs have the skills to initiate appropriate screening or diagnostic work-up to detect recurrent cancer. |  |  |  |  |
| Mani et al, 2020, USA^(12)^. | Scenario 1: - cancer recurrence screening: not at all (38%), somewhat (50%), very confident (12%)  - second cancer screening: not at all (52%), somewhat (40%), very (8%)  Scenario 2: - cancer recurrence screening: not at all 41%, somewhat 52%, very confident 7%  - second cancer screening: not at all 55%, somewhat 39%, very confident 6%. |  |  | Scenario 1: - general medical issues: somewhat (23%), very confident (77%)  - cancer-related medical issues: not at all (35%), somewhat (52%), very confident (13%).  Scenario 2: - general medical issues: not at all 2%, somewhat 20%, very confident 78%  - cancer-related medical issues: not at all 38%, somewhat 54%, very confident 8%. |  |
| McDonough et al, 2019, USA^(13)^. | 11% felt very prepared to screen for cancer recurrence. | 10% felt very prepared to manage chronic physical complications of cancer therapy.  4% felt very prepared to screen for late complications of cancer therapy. | 25% felt very prepared to manage and/or make referrals for the psychological consequences of cancer or its therapy. |  | 57% felt very prepared (31% somewhat) to provide routine age-appropriate preventive care.  51% felt very prepared (41% somewhat) to provide appropriate vaccinations. |
| Nathan et al, 2013, USA and Canada^(14)^. | Familiarity with the published guidelines was rated at a mean of 2.6 (SD=1.3). Only 9% of respondent scored their familiarity as 5 or higher.*  Respondents were most comfortable caring for survivors of HL (mean 3.7, SD 1.7), followed by ALL (mean 3.4, SD 1.6) and osteosarcoma (mean 3.3, SD 1.6). Only 33, 27, and 23 % of respondents rated their comfort as 5 or greater for caring for survivors of HL, ALL, and osteosarcoma, respectively.* | “…” | “…” |  |  |
| Nekhlyudov et al, 2014, USA^(15)^. |  | Confidence in knowledge of physical late effects in breast cancer; not at all (15%), somewhat (61%), very (23%).  Confidence in knowledge of physical late effects in colon cancer; not at all (14%), somewhat (54%), very (31%). |  |  |  |
| Park et al. 2009, USA^(16)^. |  | “Somewhat/very” vs. “not at all/a little” comfortable providing care; 87.7% vs. 12.3%.*  “Somewhat/very” vs. “not at all/a little” to evaluate long-term effects; 50.2% vs. 49.8%.* | “…” |  |  |
| Potosky et al., 2011, USA^(17)^. | 59% [56-62] strongly or somewhat agreed that PCPs have the necessary skills to provide follow-up care related to breast cancer.  75% [72-77] agreed that PCPs have the skills to initiate screening or diagnostic work-up.  58% [55-61] agreed that PCPs have the necessary skills to provide follow-up care relate to colon cancer.  74% [71-77] agreed that PCPs have the skills to initiate screening or diagnostic work-up.  40% of PCPs were "very confident" about appropriate tests for detecting recurrent disease. | 23% reported high confidence in caring for late physical effects of cancer. | 41% reported high confidence in caring for psychosocial effects of cancer. |  |  |
| Radhakrishnan et al, 2020, USA^(18)^. | High confidence in: when to refer back to the specialist (39.1%), role of neck ultrasound in long-term surveillance (36.2%), role of random thyroglobulin levels (27.2%), and when to end long-term surveillance (13.8%).  62% somewhat/strongly agreed that PCPs have the skills necessary to initiate appropriate screening to detect recurrence. |  |  |  |  |
| Roorda et al, 2013, Netherlands^(19)^. | 41% (n= 205) believed that GPs ‘had the skills necessary to take over breast cancer follow-up at an earlier stage’, whereas 20% (n= 97) felt that they ‘had the skills necessary to examine irradiated breasts to detect local recurrences and second tumours’. |  |  |  |  |
| Sima et al, 2014, USA^(20)^. | Awareness of clinical practice guidelines for the medical care of childhood cancer survivors: yes (39.6%) vs. no (60.4%). | I have had adequate training to recognize late effects of:  - chemotherapy including sterility, endocrine deficiencies, cardiomyopathy, osteoporosis, and others: strongly disagree 21.0%, somewhat disagree 36.0%, neutral 15.4%, somewhat agree 22.4%, strongly agree 5.1%.  - cancer surgeries (amputation, splenectomy etc: strongly disagree 14.7%, somewhat disagree 30.6%, neutral 18.2%, somewhat agree 30.9%, strongly agree 5.6%.  - radiation therapy (skin malignancies, local dysfunction): strongly disagree 13.7%, somewhat disagree 30.6%, neutral 17.6%, somewhat agree 33.7%, strongly agree 4.4%. |  |  |  |
| Skolarus et al, 2013, USA^(21)^. |  | Comfort level with managing:  - Urinary incontinence: very uncomfortable 12.2%, somewhat comfortable 75.6%, very comfortable 12.2%.  - Impotence: very uncomfortable 10.6%, somewhat comfortable 68.2%, very comfortable 21.2%.  - Bowel problems: very uncomfortable 10.0%, somewhat comfortable 69.8%, very comfortable 20.2%. | Comfort level with managing:  - Psychosocial concerns: very uncomfortable 9.0%, somewhat comfortable 56.5%, very comfortable 34.5%. |  |  |
| Smith et al, 2011, Canada^(22)^. | Perceived confidence in managing: - Screening for recurrence: Good=0.77, Adequate= 0.22, Low=0.01, Good or adequate overall=0.99 (n=577).  - Adjuvant hormone therapy: Good=0.30, Adequate= 0.55, Low=0.15, Good or adequate overall=0.85 (n=578) | Perceived confidence in managing:  - Treatment-related osteoporosis: Good=0.49, Adequate=0.43, Low=0.08, Overall good or adequate=0.92 (n=576).  - Treatment-induced menopause: Good=0.37, Adequate=0.51, Low=0.12, Good or adequate overall= 0.88 (n=577).  - Lymphedema: Good=0.21, Adequate=0.55, Low=0.24, Good or adequate overall=0.76 (n=576). | Perceived confidence in managing: - Anxiety or fear of recurrence: Good=0.54, Adequate=0.43, Low= 0.03, Good or adequate overall=0.97 (n=574).  - Family counselling (children or spouse): Good=0.25, Adequate=0.51, Low=0.24, Good or adequate overall=0.76 (n=575).  - Counselling on sex and body image: Good=0.30, Adequate=0.44, Low=0.26, Good or adequate overall=0.74 (n=577). |  | Perceived confidence in managing:  - Counselling on nutrition and exercise: Good=0.45, Adequate=0.44, Low=0.11, Good or adequate overall=0.89 (n=576). |
| Stephens et al, 2021, USA^(23)^. |  | 28% reported being “not confident” and an additional 17% reported being “somewhat confident” addressing the late effects of cancer treatment. |  | 24.1% reported being “somewhat” or “not confident” addressing chronic comorbidities. |  |
| Suh et al, 2014, USA^(24)^. | Internists reported being generally unfamiliar with available surveillance guidelines for CCSs. Only 12.0% stated that they felt at least “somewhat familiar” with available guidelines (Likert score ≥5). | Internists reported being “somewhat uncomfortable” caring for CCSs. Only 36.9%, 27.0%, and 25.0% of respondents indicated that they were “somewhat comfortable” or “comfortable” (Likert score ≥5) caring for Hodgkin lymphoma, acute lymphoblastic leukemia, and osteosarcoma survivors, respectively. |  |  |  |
| Virgo et al, 2013, USA^(25)^. | Self-ascribed knowledge of cancer-related follow-up care for breast and colon cancer survivors; low 47.0% (n = 65.896), medium 36.0% (n = 50.481), high 16.9% (n = 23.774) and missing 0.1% (n = 202).* | “…” | “…” |  |  |
| Walter et al, 2015, UK^(26)^. |  | Knowledge of associations between types of cancer treatment and reduced bone health (hormone therapy n=417, 83%; chemotherapy n=338, 68%; radiotherapy n=290, 58%).  Knowledge of associations with reduced cardiovascular health (hormone therapy n=267, 53%; chemotherapy n=249, 50%; radiotherapy n=200, 40%).  Confidence in management of treatment-related side effects: not at all (5%), somewhat (65%), very (30%). | Confidence in management of psychological symptoms: not at all (3%), somewhat (50%), very (47%).  Confidence in advice concerning work and/or finances: not at all (23%), somewhat (58%), very (19%). |  | Confidence in lifestyle health care: not at all (1%), somewhat (34%), very (65%). |

* The result could be mapped to multiple domains (indicated by “…”).

**Supplementary file 3 – Other related outcomes.**

| **Author, year, country** | **Other related outcomes** | **Results** |
| --- | --- | --- |
| Bober et al, 2009, USA^(4)^. | Barriers to care. | Commonly endorsed barriers were lack of standards of care (52.5%, 95% CI 45.7-59.3), inadequate preparation/formal training (47.2%, 95% CI 40.4-54.1), limited access to mental health referrals (45.7%, 95% CI 38.9-52.5), lack of time to adequately address cancer survivorship issues (42.4%, 95% CI 35.7-49.3), inadequate access to patients’ cancer treatment history (36.1%, 95% CI 29.7-42.8), patient anxiety or fears about health (28.8%, 95% CI22.9-35.3), lack of practical experience in caring for cancer survivors (22.4%, 95% CI 16.9-27.9), limited access to cancer specialists when needed (10.5%, 95% CI 6.8-15.3), limited access to noncancer specialists such as cardiac or endocrine specialists (7.8%, 95% CI 4.6-12.1), patient reluctance to discuss previous cancer history (2.3%, 95% CI 0.8-5.3). |
| Chow et al, 2017, Canada^(7)^. | Barriers to care. | Common barriers to treatment were cost/coverage of medications (n = 72, 54.1%), patient misuse or abuse of opioids (n = 68, 51.1%), patient non-compliance (n = 62, 46.6%), lack of knowledge of evidence-based clinical practice guidelines and treatments (n = 57, 42.9%), concern about the ability of patient or caregiver to follow (n = 57, 42.9%), uncertainty about how to execute a stepwise approach to pain management (n = 40, 30.1%) and uncertainty about which medications to prescribe (n = 34, 25.6%). Lack of time was reported (n = 26, 19.5%) and other barriers (n = 16, 12.0%). Seven physicians (5.3%) reported that they do not encounter any barriers. |
| Chow et al, 2017, Canada^(7)^. | Educational needs. | Causes of chronic pain related to cancer treatment (n = 75, 56.4%), classification/characteristics of chronic pain (n= 60, 45.1%), treatment guidelines (n = 98, 73.7%), pain assessment (n = 61, 45.9%), treatment goals (n = 64, 48.1%), treatment options (n = 86, 64.7%), effectiveness of medications (n = 54, 40.6%), tolerability of medications (n = 51, 38.3%), switching treatment (n = 64, 48.1%), monitoring treatment (n = 50, 37.6%), non-pharmalogical treatment options (n = 83, 62.4%), value of psychotherapy or cognitive behavioral therapy (n = 75, 56.4%), when to refer a patient to a specialist (n = 57, 42.9%) and other (n = 8, 6.0%). |
| González Carnero et al, 2013, Spain^(10)^. | Educational needs. | The biggest need for training focuses on emergency care in terminal patients (mean 3.77, SD 1.04) and management of complications of cancer treatment (mean 3.71, SD 0,96). On the other hand, training on pain management (mean 2.89, SD 1.15), respiratory problems (mean 2.88, SD 1.10), and digestive problems (mean 2.66, SD 1.02) are less needed. |
| González Carnero et al, 2013, Spain^(10)^. | Barriers to care (involvement in care). | The most cited reasons for lack of involvement in care were lack of experience (58.1%), followed by lack of time (47.7%), lack of training (46.5%) and lack of confidence (45.9%). |
| Mani et al, 2020, USA^(12)^. | Barriers to care. | Physician related barriers to care were; lack of resources to facilitate care (69%), lack of awareness of psychosocial needs (65%), lack of awareness of guidelines (55%) and insufficient time (65%). Patient related barriers were; unclear who to approach for care (77%), preference to follow-up with oncologist (66%), inadequate communication with oncologist (52%) and insufficient reimbursement (43%). |
| McDonough et al, 2019, USA^(13)^. | Barriers to care. | The most frequently endorsed barriers were uncertainty about care delegation(73%) and lack of training (72%). Lack of access to adequate patient information, such as treatment history and an SCP delineating screening needs, were also commonly reported (33% and 54%, respectively). More than one half (56%) reported that a patient’s preference to have his or her oncologist manage cancer-related issues served as a barrier. One third (34%) reported feeling that oncologists preferred to manage cancer-related issues. |
| Park et al. 2009, USA^(16)^. | Barriers to care. | Perceived barriers to survivorship care (no/small vs. moderate/big problem): lack of practical experience in caring for cancer survivors (77.6% vs 22.4%), lack of time to adequately address survivorship issues (57.6% vs. 42.4%), inadequate preparation/formal training around survivorship issues (52.8% vs. 47.2%), limited access to cancer specialists (89.5% vs. 10.5%), limited access to non-cancer specialists (92.2% vs. 7.8%), inadequate access to cancer treatment history (63.9% vs. 36.1%), lack of standards of care for adult survivors (47.5% vs. 52.5%), patient anxiety or fears about health (71.2% vs. 28.8%), patient reluctance to discuss cancer history (97.7% vs. 2.3%), limited access to mental health referrals for survivors (54.3% vs. 45.7%). |
| Roorda et al, 2013, Netherlands^(19)^. | Recommendations for care. | GP recommendations for improving current and future primary care-based follow-up: local agreements with specialists on follow-up policy, active discharge of patients by specialists, adequate supply of written discharge information, quick referral of patients to the breast cancer clinic when necessary, education and training of GPs in follow-up care and development of administrative tools to support the organisation of follow-up in general practice. |
| Virgo et al, 2013, USA^(25)^. | Barriers to care. | - Total patient-related barrier score; low (n = 108.187, 82.1%), high (n = 23.523, 17.9%).  - Total physician-related barrier score; low (n = 108.863, 82.7%), high (n = 22.846, 17.3%).  - One of the physician-related barriers, is the following question "I don't have adequate knowledge or training to manage my patients' problems". PCPs answered with; often/always (n = 4.825, 3.8%), sometimes (n = 54.058, 42.6%) or never/rarely (n = 67.964, 53.6%). |
| Walter et al, 2015, UK^(26)^. | Educational needs. | Areas of desired further education: - Management of cardiovascular consequences following treatment (n = 430, 86%) - Management of bone consequences following treatment (n = 411, 82%) - Management of treatment-related side effects (n = 381, 76%) - Management of psychological symptoms (n = 260, 52%) - Advice concerning work and/or finances (n = 180, 36%) - Lifestyle health care (n = 116, 23%). |

**Supplementary file 4 – Qualitative outcomes according to the cancer survivorship care domains.**

| **Author, year, country** | **Results** |
| --- | --- |
| Duffey-Lind et al, 2006, USA^(27)^. | Physicians reported problems in the following areas: (1) missing information, (2) lack of knowledge, (3) sparse number of childhood cancer survivors in primary care practice, and (4) time constraints. Physicians felt that they needed to be educated regarding their follow-up plan including potential late effects of therapies received. When asked what would be the most helpful to them, all 3 physicians stated that they would like to have general information on pediatric oncology and treatment, in addition to individual patient information. |
| Dawes et al, 2015, USA^(8)^. | Particular concerns included the timing of transition (ie, how long after active treatment); unfamiliarity with the management of endocrine therapy; and confusion regarding who is responsible for providing certain aspects of care, particularly emotional and psychological support. Strategies to improve the transition included additional training on current clinical guidelines for breast cancer survivorship care and the provision of survivorship care plans on transfer of care. |
| Fox et al, 2021, Australia^(28)^. | It was suggested that GPs had the required survivorship skills. Indeed, with some guidance and a framework, they would be in a position to provide adequate care. - GP04 stated: "Treatments have changed so much. When I get the letters and specialists are describing new treatments that it is quite difficult for the GP to keep up to date with what the latest treatments are, and we may not always be confident in, ’What are the adverse effects for this drug?’ and so on." - GP01 stated: "A lack of knowledge and experience in that area are the biggest challenges and the oncology side of things is quite specialised, so having enough understanding that you can still support the patient can be difficult, but all that is about education and experience." |
| Heins et al, 2018, Netherlands^(29)^. | GPs were confident providing this type of care, as the protocol provided clear criteria for referral to the urologist. Some perceived scheduling and keeping track of follow-up appointments as challenging; as they only had a few patients participating, it was not integrated into their routine procedures. They could always reach a urologist if necessary. |
| Margariti et al, 2020, UK^(30)^. | Nine participants considered themselves well-prepared for providing follow-up care. This was grounded in their wide experience of managing different cancers and chronic conditions other than cancer. This confidence was, to a degree, dependent on the accessibility of referral back to specialist care if necessary. However, this, in turn, depended on the hospital discharging the prostate cancer survivors in a stable condition and with a treatment plan. Participants expressed their concerns involving follow-up and recall and test interpretation. They worried about the possibility of “losing” some patients between their follow-up appointments as they would not be able to provide them with reminders. In addition, they felt that their limited knowledge concerning the interpretation of PSA results could have a negative impact on the patients. Several expressed a desire for training and updating in order to improve their and their nurses' skills and knowledge in providing services to prostate cancer patients. |
| Radhakrishnan et al, 2019, USA^(31)^. | PCPs had general knowledge about prostate cancer survivorship care including assessing for treatment side effects and managing complications (eg, erectile dysfunction) and monitoring for recurrence (eg, serial PSA testing). However, knowledge barriers to survivorship care were also noted. PCPs reported lack of familiarity with or not receiving survivorship care plans. PCPs also endorsed having procedural knowledge about how to deliver survivorship care. PCPs endorsed having professional confidence (an individual's belief in his or her repertoire of skills, and ability especially as it is applied to a task or set of tasks) in handling many aspects of follow-up care for their patients and feeling comfortable doing so.  - One PCP noted, “… I think we try to manage them … most of the time probably. Primary Care does the majority of managing of the symptoms … and then for the ones that are really refractory we end up sending them back to urology, but I do feel kind of responsible for a pretty broad range.” |
| Sarfo et al, 2022, Netherlands^(32)^. | Some GPs stated that cancer patients currently receive enough guidance from health providers in the hospital and they do not consider work guidance or RTW to be their task. Furthermore, most GPs described they do not wish to get involved with patients’ legal rights to illness benefits. This is due to not having sufficient knowledge or experience in work regulations and legislation associated with these benefits and therefore feeling at risk of being involved in a conflict situation between employers and patients. As such, GPs described being hesitant with offering work guidance in the form of a direct recommendation to return to work. Rather, they stated that the importance of work and advice regarding work is discussed in the context of restoring patients’ overall well-being and balance, and generally as a part of aftercare and psychosocial guidance. |
| Signorelli et al, 2019, Australia and New Zealand^(33)^. | PCPs felt confident providing care to adult cancer survivors, whereas only 54% of PCPs reported feeling confident providing care to CCS. Many (63.2%) PCPs reported feeling confident that they specifically understood survivors’ current and future health needs, generally portrayed as having a simple or “basic idea” (male PCP, practicing 57 years) of survivors’ needs. Most PCPs (79%) reported feeling comfortable assuming full responsibility for the follow-up of CCS, if the survivor stopped attending survivorship clinics. Yet, PCPs’ confidence and willingness to assume full responsibility for CCS appeared to depend on various factors, including being part of a team or having clear direction from oncologists. PCPs’ confidence in survivors’ future health needs was also somewhat superficial, with one PCP commenting about their patient: “If he’s not complaining of anything I’m confident that there’s nothing wrong” (male PCP, practicing 27 years). Low confidence appeared to be related to poor knowledge about the specific protocols recommended for each survivor, particularly “the kind of routine follow-up I should do or what sort of anticipatory care [is needed]” (male PCP, practicing 25 years). Qualitatively, PCPs attributed their lack of specific knowledge, and therefore confidence, to their inexperience and the few CCS they had seen in their career compared with adult cancer survivors. PCPs’ confidence was not associated with PCPs’ years of experience practicing (t(46)=−0.808, p=.808) and the number of CCS they had cared for in their career (t(44)=−0.699, p=.488). |
| Stephens et al, 2021, USA^(23)^. | Most participants (n=8) reported a lack of formal training specific to survivorship care, especially for the care of geriatric patients. A common response when asked about training was along the lines of, “As far as survivorship, I would say zero” or “I don’t have any specific training.” When asked specifically about their residency training, participants indicated a lack of education related to the effects of cancer treatment and survivorship. One participant said, “my residency did not deal as much with the effects of actually treating.” Another said, “I never, to my regret, actually had a specific training as a primary care doctor on how to deal with malignancies and the co-care of patients. ”Some participants (n=3) did indicate having completed a few oncology rotations during their training, but these individuals also reported not being as “engaged as I maybe could have been because I knew I wasn’t going to go into [that field].” A few participating physicians (n= 3) had extensive experience related to cancer care. Experiences included completing a master’s degree, going to yearly conferences, and serving on an American Cancer Society board. It was most common, however, for PCPs to report that they “learn as I go, ”“research what I need to,” or have “cobbled together some training ”throughout their career. The Key Findings were summarized as: - lack of geriatric-specific training - lack of training related to cancer treatment and survivorship - most PCPs educate themselves on survivorship care |
| Thamm et al, 2022, Australia^(34)^. | GPs identified challenges in addressing the financial burden of cancer patients. These challenges were particularly related to their knowledge of diagnostic, treatment, and specialist costings. ‘This was related to the fact that ‘anyone can charge anything’ (GP17): I unfortunately wouldn’t be informed as to how much out-of-pocket expenses they might have ... Broaching a topic that you have no knowledge about, is tantamount to opening a Pandora’s Box. (GP14) GPs also highlighted that, while for some cancers managing financial concerns may be achievable, the complexity and heterogeneity of cancer care made it a challenge to address FT: For prostate, breast, melanoma even, we do have a sense of what that [pathway] might look like, but for cancers which are less common I think it is harder for us to know ... to give meaningful financial counselling. (GP4) GPs identified that information about costs was learnt ‘along the way’ and from patient-reported experiences. Many felt that additional cost information would improve their knowledge as specialists did not always provide clear information about this: I think it probably would be interesting to have a bit more knowledge when people are trying to make those early decisions about if they go private, what are the costs to be expected. (GP6) In regards to treatment pathway facilitation: some felt their experience and local knowledge put them in the position to steer a patient in a certain direction. |
| Vos et al, 2022, Netherlands^(35)^. | Overall, GPs expressed that cancer survivorship care is not complex and dealing with cancer repercussions is already part of their current work. Nevertheless, there was a common request among GPs for additional education regarding post treatment symptoms, side-effects and how to handle them after both 1- and 5-years of care. The disadvantages for the GPs centered around their lack of experience and routine with this type of care. Delivering colon cancer survivorship care in primary care was not deemed difficult. However, GPs were not able to remember the follow-up schedule and had to look up the schedule during each consultation. Time was lost figuring out the protocol and contacting secondary care physicians. This was mentioned after both 1- and 5-years of care. Most GPs felt confident in delivering survivorship care, though some doubted their experience with this type of care, knowledge and capabilities. “In a general practice there are not that many patients with colon cancer who are in this phase. Even if you were to include all the patients from my practice, then it still wouldn’t be sufficient to gain any real routine” (male, 5 years of care). |

**References**

1. Moola S, Munn Z, Tufanaru C, Aromataris E, Sears K, Sfetcu R, Currie M, Qureshi R, Mattis P, Lisy K, Mu P-F. Chapter 7: Systematic reviews of etiology and risk . In: Aromataris E, Munn Z (Editors). JBI Manual for Evidence Synthesis. JBI, 2020. Available from <https://synthesismanual.jbi.global>.

2. Lockwood C, Munn Z, Porritt K. Qualitative research synthesis: methodological guidance for systematic reviewers utilizing meta-aggregation. Int J Evid Based Healthc. 2015;13(3):179–187.

3. Berry-Stoelzle M, Parang K, Daly J. Rural Primary Care Offices and Cancer Survivorship Care: Part of the Care Trajectory for Cancer Survivors. *Health Serv Res Manag Epidemiol*. 2019;**6**:2333392818822914.

4. Bober SL, Recklitis CJ, Campbell EG, et al. Caring for cancer survivors: a survey of primary care physicians. *Cancer*. 2009;**115**(18 Suppl):4409-18.

5. Cheung WY, Aziz N, Noone A-M, et al. Physician preferences and attitudes regarding different models of cancer survivorship care: A comparison of primary care providers and oncologists. *Journal of Cancer Survivorship*. 2013;**7**(3):343-54.

6. Chou C, Hohmann NS, Hastings TJ, et al. How comfortable are primary care physicians and oncologists prescribing medications for comorbidities in patients with cancer? *Res Social Adm Pharm*. 2020;**16**(8):1087-94.

7. Chow R, Saunders K, Burke H, et al. Needs assessment of primary care physicians in the management of chronic pain in cancer survivors. *Support Care Cancer*. 2017;**25**(11):3505-14.

8. Dawes AJ, Hemmelgarn M, Nguyen DK, et al. Are primary care providers prepared to care for survivors of breast cancer in the safety net? *Cancer*. 2015;**121**(8):1249-56.

9. Fidjeland HL, Brekke M, Vistad I. General practitioners' attitudes toward follow-up after cancer treatment: A cross-sectional questionnaire study. *Scand J Prim Health Care*. 2015;**33**(4):223-32.

10. Gonzalez Carnero R, Sanchez Nava JG, Canchig Pilicita FE, et al. Training needs in the care of oncological patients. *Medicina Paliativa*. 2013;**20**(3):103-10.

11. Klabunde CN, Han PK, Earle CC, et al. Physician roles in the cancer-related follow-up care of cancer survivors. *Fam Med*. 2013;**45**(7):463-74.

12. Mani S, Khera N, Rybicki L, et al. Primary Care Physician Perspectives on Caring for Adult Survivors of Hematologic Malignancies and Hematopoietic Cell Transplantation. *Clin Lymphoma Myeloma Leuk*. 2020;**20**(2):70-7.

13. McDonough AL, Rabin J, Horick N, et al. Practice, Preferences, and Practical Tips From Primary Care Physicians to Improve the Care of Cancer Survivors. *J Oncol Pract*. 2019;**15**(7):e600-e6.

14. Nathan PC, Daugherty CK, Wroblewski KE, et al. Family physician preferences and knowledge gaps regarding the care of adolescent and young adult survivors of childhood cancer. *Journal of Cancer Survivorship*. 2013;**7**(3):275-82.

15. Nekhlyudov L, Aziz NM, Lerro C, Virgo KS. Oncologists' and primary care physicians' awareness of late and long-term effects of chemotherapy: implications for care of the growing population of survivors. *J Oncol Pract*. 2014;**10**(2):e29-e36.

16. Park ER, Bober SL, Campbell EG, et al. General internist communication about sexual function with cancer survivors. *Journal of General Internal Medicine*. 2009;**24**(Suppl 2):S407-S11.

17. Potosky AL, Han PK, Rowland J, et al. Differences between primary care physicians' and oncologists' knowledge, attitudes and practices regarding the care of cancer survivors. *J Gen Intern Med*. 2011;**26**(12):1403-10.

18. Radhakrishnan A, Reyes-Gastelum D, Gay B, et al. Primary Care Provider Involvement in Thyroid Cancer Survivorship Care. *J Clin Endocrinol Metab*. 2020;**105**(9):e3300-e6.

19. Roorda C, Berendsen AJ, Haverkamp M, et al. Discharge of breast cancer patients to primary care at the end of hospital follow-up: a cross-sectional survey. *Eur J Cancer*. 2013;**49**(8):1836-44.

20. Sima JL, Perkins SM, Haggstrom DA. Primary care physician perceptions of adult survivors of childhood cancer. *J Pediatr Hematol Oncol*. 2014;**36**(2):118-24.

21. Skolarus TA, Holmes-Rovner M, Northouse LL, et al. Primary care perspectives on prostate cancer survivorship: implications for improving quality of care. *Urol Oncol*. 2013;**31**(6):727-32.

22. Smith SL, Wai ES, Alexander C, Singh-Carlson S. Caring for survivors of breast cancer: perspective of the primary care physician. *Curr Oncol*. 2011;**18**(5):e218-e26.

23. Stephens C, Klemanski D, Lustberg MB, et al. Primary care physician's confidence and coordination regarding the survivorship care for older breast cancer survivors. *Support Care Cancer*. 2021;**29**(1):223-30.

24. Suh E, Daugherty CK, Wroblewski K, et al. General internists' preferences and knowledge about the care of adult survivors of childhood cancer: a cross-sectional survey. *Ann Intern Med*. 2014;**160**(1):11-7.

25. Virgo KS, Lerro CC, Klabunde CN, et al. Barriers to breast and colorectal cancer survivorship care: perceptions of primary care physicians and medical oncologists in the United States. *J Clin Oncol*. 2013;**31**(18):2322-36.

26. Walter FM, Usher-Smith JA, Yadlapalli S, Watson E. Caring for people living with, and beyond, cancer: an online survey of GPs in England. *Br J Gen Pract*. 2015;**65**(640):e761-e8.

27. Duffey-Lind EC, O'Holleran E, Healey M, et al. Transitioning to survivorship: a pilot study. *J Pediatr Oncol Nurs*. 2006;**23**(6):335-43.

28. Fox J, Thamm C, Mitchell G, et al. Cancer survivorship care and general practice: A qualitative study of roles of general practice team members in australia. *Health & Social Care in the Community*. 2021:No-Specified.

29. Heins M, Korevaar J, Van Dulmen S, et al. Feasibility and acceptability of follow-up care for prostate cancer in primary care. *European Journal of Cancer*. 2017;**72**(Supplement 1):S186.

30. Margariti C, Gannon KN, Walsh JJ, Green JSA. GP experience and understandings of providing follow-up care in prostate cancer survivors in England. *Health Soc Care Community*. 2020;**28**(5):1468-78.

31. Radhakrishnan A, Henry J, Zhu K, et al. Determinants of quality prostate cancer survivorship care across the primary and specialty care interface: Lessons from the Veterans Health Administration. *Cancer Med*. 2019;**8**(5):2686-702.

32. Sarfo MC, Bertels L, Frings-Dresen MHW, et al. The role of general practitioners in the work guidance of cancer patients: views of general practitioners and occupational physicians. *J Cancer Surviv*. 2022:1-9.

33. Signorelli C, Wakefield CE, Fardell JE, et al. The Role of Primary Care Physicians in Childhood Cancer Survivorship Care: Multiperspective Interviews. *Oncologist*. 2019;**24**(5):710-9.

34. Thamm C, Fox J, Hart NH, et al. Exploring the role of general practitioners in addressing financial toxicity in cancer patients. *Support Care Cancer*. 2022;**30**(1):457-64.

35. Vos JAM, de Best R, Duineveld LAM, et al. Delivering colon cancer survivorship care in primary care; a qualitative study on the experiences of general practitioners. *BMC Prim Care*. 2022;**23**(1):13.
